# Impact of everolimus-related interstitial lung disease on subsequent treatment in patients with metastatic breast cancer

**DOI:** 10.1093/oncolo/oyag082

**Published:** 2026-03-14

**Authors:** Hikari Kiyohara, Chihiro Kondoh, Akira Hirota, Nobuyuki Takahashi, Misao Fukuda, Shota Kusuhara, Takehiko Nakao, Hiromichi Nakajima, Chikako Funasaka, Kenichi Harano, Nobuaki Matsubara, Yoichi Naito, Ako Hosono, Naoki Niikura, Toru Mukohara

**Affiliations:** Department of Medical Oncology, National Cancer Center Hospital East, Kashiwa, 277-8577, Japan; Department of Breast Oncology, Tokai University School of Medicine, Isehara, Kanagawa, 259-1193, Japan; Department of Medical Oncology, National Cancer Center Hospital East, Kashiwa, 277-8577, Japan; Department of Medical Oncology, Saitama Cancer Center, Kita-adachi-gun, Saitama, 362-0806, Japan; Department of Medical Oncology, National Cancer Center Hospital East, Kashiwa, 277-8577, Japan; Department of Medical Oncology, National Cancer Center Hospital East, Kashiwa, 277-8577, Japan; Department of Medical Oncology, National Cancer Center Hospital East, Kashiwa, 277-8577, Japan; Department of Medical Oncology, National Cancer Center Hospital East, Kashiwa, 277-8577, Japan; Department of Medical Oncology, National Cancer Center Hospital East, Kashiwa, 277-8577, Japan; Department of Medical Oncology, National Cancer Center Hospital East, Kashiwa, 277-8577, Japan; Department of Experimental Therapeutics, National Cancer Center Hospital East, Kashiwa, 277-8577, Japan; Department of General Internal Medicine, National Cancer Center Hospital East, Kashiwa, 277-8577, Japan; Department of Medical Oncology, National Cancer Center Hospital East, Kashiwa, 277-8577, Japan; Department of Experimental Therapeutics, National Cancer Center Hospital East, Kashiwa, 277-8577, Japan; Department of Medical Oncology, National Cancer Center Hospital East, Kashiwa, 277-8577, Japan; Department of Experimental Therapeutics, National Cancer Center Hospital East, Kashiwa, 277-8577, Japan; Department of Medical Oncology, National Cancer Center Hospital East, Kashiwa, 277-8577, Japan; Department of Medical Oncology, National Cancer Center Hospital East, Kashiwa, 277-8577, Japan; Department of Experimental Therapeutics, National Cancer Center Hospital East, Kashiwa, 277-8577, Japan; Department of General Internal Medicine, National Cancer Center Hospital East, Kashiwa, 277-8577, Japan; Department of Medical Oncology, National Cancer Center Hospital East, Kashiwa, 277-8577, Japan; Department of Pediatric Oncology, National Cancer Center Hospital East, Kashiwa, 277-8577, Japan; Department of Breast Oncology, Tokai University School of Medicine, Isehara, Kanagawa, 259-1193, Japan; Department of Medical Oncology, National Cancer Center Hospital East, Kashiwa, 277-8577, Japan

**Keywords:** everolimus, interstitial lung disease, subsequent treatment, breast cancer, S-1, paclitaxel

## Abstract

**Background:**

Everolimus is an option for second-line or later use in combination with endocrine therapy for hormone receptor-positive breast cancer. An important everolimus-associated adverse event is drug-related interstitial lung disease (ILD), reported to occur in 16.0% of individuals in the BOLERO-2 trial, and 23.5% of those in the Asian subgroup. We aimed to examine the impact of everolimus-related ILD (e-rILD) on the risk of drug-related ILD during subsequent anti-cancer treatment.

**Patients and methods:**

We retrospectively studied the medical records of patients with metastatic breast cancer treated with everolimus plus endocrine therapy from January 2013 to March 2022, at the National Cancer Center Hospital East. We evaluated e-rILD grade using the Common Terminology Criteria for Adverse Events version 5.0.

**Results:**

A total of 115 cases were treated with everolimus; 33 (28.7%) patients developed e-rILD of any grade, including 5 (15.2%) with grade ≥3 and 2 with grade 5. Management approaches for e-rILD included dose continuation, dose interruption, prednisolone 0.5-1.0 mg/kg, and steroid pulse. The proportions of patients who received subsequent treatment were similar between the e-rILD and no e-rILD groups (73.2% [60/82] vs 81.8% [27/33], respectively; *P* = .567). Subsequent therapies were generally similar between the 2 groups. Drug-related ILD during regimens following everolimus occurred in one patient (3.7%) with e-rILD and 2 patients (3.3%) without e-rILD (*P* = .930; odds ratio, 1.12; 95% CI, 0.10-12.86).

**Conclusion:**

e-rILD may not influence the choice of following treatment or the development of drug-related ILD during subsequent treatments.

Implications for Practice Everolimus remains an important component of treatment for metastatic ER+/HER2- breast cancer, yet concern about interstitial lung disease (ILD) often leads clinicians to discontinue therapy and hesitate to initiate further systemic treatment. In this study, most everolimus-related ILD (e-rILD) events were mild, reversible, and manageable with appropriate intervention and did not appear to preclude the use of standard systemic regimens immediately after everolimus. Within the limits of this retrospective analysis, these findings support the feasibility of carefully monitored post-everolimus treatment and may help clinicians make rational sequencing decisions following e-rILD without unnecessarily restricting subsequent options.

## Introduction

Breast cancer is the most frequently diagnosed cancer and leading cause of cancer-related death in women worldwide. According to Global Cancer Observatory 2022 statistics, 23.8% of cancer cases in women and 15.4% of cancer deaths are attributable to breast cancer.^1^ Hormone receptor-positive (HR+)/human epidermal growth factor receptor 2-negative (HER2-) breast cancer is the most frequently diagnosed subtype of breast cancer, and metastatic or recurrent cases in the absence of visceral crisis are treated with endocrine therapy alone or in combination with molecularly targeted agents.

Everolimus is one of a number of mammalian target of rapamycin (mTOR) inhibitors that inhibit mTOR signaling within the PI3K–AKT–mTOR pathway, which is involved in acquired resistance to endocrine therapy in patients with breast cancer.^2^ In the BOLERO-2 trial, everolimus plus exemestane significantly improved progression-free survival (PFS) compared with exemestane plus placebo in patients with HR+/HER2- metastatic breast cancer that had advanced after nonsteroidal aromatase inhibitor therapy (median PFS, 7.8 months in the everolimus group vs 3.2 months in the placebo group, *P* < .0001).^3^ In a phase II trial, fulvestrant plus everolimus significantly improved median PFS compared with fulvestrant alone in patients with aromatase inhibitor-resistant metastatic breast cancer (10.3 months vs 5.1 months; hazard ratio [HR] 0.61, 95% CI 0.40-0.92; *P* = .02).^4^

Although the BOLERO-2 trial failed to demonstrate a benefit in overall survival (OS) or quality of life, and the improvement in PFS was suggested to be influenced by informative censoring, current guidelines recommend everolimus plus exemestane as an option for second-line or later endocrine therapy.^5–8^ Furthermore, in the phase III evERA trial,^9^ among patients with *ESR1*-mutated disease previously treated with CDK4/6 inhibitors, giredestrant plus everolimus achieved a longer median PFS of 9.99 months (95% CI, 8.08-12.94) compared with 5.45 months (95% CI, 3.75-5.62) with everolimus plus standard endocrine therapy (HR, 0.38; 95% CI, 0.27-0.54; *P* < .0001); however, OS data were immature at the time of analysis. These results suggest that everolimus-based regimens will continue to play an important role in later-line therapy for HR+/HER2- advanced breast cancer and renewed interest in everolimus.

Frequently occurring everolimus-associated adverse events include stomatitis, asthenia, skin rashes, and abnormalities in glucose metabolism. Another significant adverse event is drug-related interstitial lung disease (ILD), which was reported in 16.0% of cases in the BOLERO-2 study.^10^ There are ethnicity-related differences in the incidence of everolimus-related ILD (e-rILD), with a reportedly higher incidence in Asian populations than in patients from other regions.^10^ It is unclear whether the development of e-rILD is associated with an increased risk of developing drug-related ILD with subsequent anti-cancer treatment. This study aimed to examine the impact of e-rILD on the choice of subsequent treatments and the later incidence of ILD in real-world clinical practice.

## Patients and methods

### Study design and patient population

This was a single-center, retrospective study. We performed a medical chart review of all consecutive patients with advanced breast cancer who underwent everolimus plus endocrine therapy from January 1, 2013 to March 31, 2022, at the National Cancer Center Hospital East. Patients received everolimus at a dose of 10 mg once daily. Dose reduction, when required, was defined as a 1-step reduction to 5 mg once daily. Before treatment initiation, all patients underwent baseline imaging with contrast-enhanced computed tomography (CT) from the neck to the pelvis, including evaluation of the lungs; however, high-resolution CT was not routinely performed. After treatment initiation, follow-up CT scans were performed at regular intervals of every 8-12 weeks to assess treatment responses. Additional imaging evaluations were conducted at the discretion of the treating physician when new clinical symptoms, including respiratory symptoms, developed. A diagnosis of ILD was determined based on radiological assessments reported by a radiologist, with careful exclusion of other causes, such as infectious pneumonia, heart failure, or lymphangitic carcinomatosa; serum β-d-glucan and plasma brain natriuretic peptide were measured in most cases. Asymptomatic cases of ILD incidentally detected on CT were also included. Cases in which drug-related ILD could not be excluded after this evaluation were classified as e-rILD. The ILD grade was assessed using the Common Terminology Criteria for Adverse Events version 5.0. We compared the clinical courses of patients who experienced e-rILD with those of patients who did not. The definition of the next regimen after everolimus was the regimen administered after both everolimus and the concomitant endocrine therapy drugs were terminated.

### Ethics

This study was approved by the Institutional Review Board of the National Cancer Center (approval number: 2017-431) and conducted in compliance with the Ethical Guidelines for Epidemiological Research in Japan. The requirement for written informed consent was waived by the Institutional Review Board.

### Statistical analysis

Patient background data, outcomes after everolimus treatment, and subsequent regimens were compared between patients with and without e-rILD using the χ^2^ or Fisher’s exact test. Odds ratios (ORs) were calculated for ILD onset during the treatment regimen after everolimus. OS was calculated from the initiation of everolimus to death from any cause. OS from subsequent therapy initiation was defined as the time from the initiation of the first systemic treatment administered after the discontinuation of everolimus to death from any cause. Time to treatment failure (TTF) was defined as the time from the initiation of everolimus to treatment discontinuation for any reason, including disease progression, adverse events, or death. Patients without events were censored at the date of the last follow-up. The median follow-up time was calculated using the reverse Kaplan–Meier method, defined as the time from the initiation of everolimus to the last confirmed date of contact, disease progression, or death. We analyzed OS and TTF using the Kaplan–Meier method. Hazard ratios and 95% CIs for OS were estimated using the Cox regression model. All *P*-values were 2-tailed, with *P* < .05 considered statistically significant. Statistical analyses were performed with EZR version 1.60 (Saitama Medical Center, Jichi Medical University), a graphical user interface for R (The R Foundation for Statistical Computing).

## Results

### Patient characteristics

From January 2013 to March 2022, all patients (*n* = 115) who received everolimus plus endocrine therapy during the study period were included in the study. Of those patients, 33 (28.7%) developed e-rILD, and 82 (71.3%) did not. At the data cut-off point (June 30, 2022), the median follow-up period was 33.5 months (range 3.9-94.3) for patients with e-rILD and 35.7 months (range 0.5-78.5) for those without e-rILD, with no significant difference between the 2 groups (Table 1). The median age was 66 years for those with e-rILD and 64 years for those without e-rILD. The median treatment line of everolimus was third for those with e-rILD and fourth for those without e-rILD. Most of the endocrine therapies combined with everolimus were aromatase inhibitors in both groups. Previous treatment with CDK4/6 inhibitors had occurred for 48.5% of patients with ILD and 42.7% without ILD (Table 1). Among patients who developed ILD, the most common comorbidities were hypertension in 7 cases (21.2%), diabetes mellitus in 6 cases (18.2%), and rheumatoid arthritis in 1 case (3.0%). None of the patients had pre-existing ILD as an underlying condition. Eleven (33.3%) patients who developed ILD had a history of smoking.

**Table 1:** Patient characteristics.

	Patients treated with everolimus		
	With e-rILD (*n* = 33)	Without e-rILD (*n* = 82)	*P*-value
**Median follow-up (months) (95% CI)**	33.5 (17.2-not reached)	35.7 (28.7-61.2)	.965
**Age (year), median (range)**	66 (47-77)	64 (42-81)	.110
**Sex (female), *n* (%)**	33 (100)	81 (100)	—
**Number of previous systemic treatments (range)**	2 (1-7)	3 (0-13)	.015
**Combined endocrine therapy, *n***			
** Aromatase inhibitor**	32	81	
** Tamoxifen**	1	0	
** Aromatase inhibitor followed by tamoxifen**	0	1	
**History of CDK4/6 inhibitor therapy, *n* (%)**	16 (48.5)	35 (42.7)	.571

Abbreviations: CDK, cyclin-dependent kinase; e-rILD, everolimus-related interstitial lung disease; *n*, number.

### Details of e-rILD

The median time from the start of everolimus to the onset of e-rILD was 64 d (range, 40-252 d). ILD severity was grade 1 or 2 in 28 cases (84.9%), grade 3 in 3 cases (9.1%), and grade 5 in 2 cases (6.1%). One of the grade 5 patients had a high level of β-d-glucan, which indicates that the patient may have had pneumocystis pneumonia, but e-rILD could not be excluded. The initial management of e-rILD was close monitoring only while continuing everolimus in one grade 1 case, dose interruption only in 9 cases, dose interruption with prednisolone 0.5-1.0 mg/kg/d in 20 cases, and dose interruption with steroid pulse (methylprednisolone 1000 mg/d) for 3 days in 3 cases. Everolimus was restarted in 12 of 33 cases (36.4%) with e-rILD after recovering to grade 0 or 1. Of these 12 cases, 2 resumed everolimus at the original dose, 3 resumed treatment at the same dose because e-rILD had developed while they were already receiving everolimus with a one-step dose reduction because of other adverse events, and the remaining 7 cases resumed everolimus with a one-step dose reduction after the onset of e-rILD. After restarting everolimus, one patient experienced relapse to grade 2 e-rILD, but recovered to grade 1 after treatment with prednisolone at 0.5 mg/kg/d.

### Choice of post-everolimus therapy and outcome

After treatment with everolimus was terminated, 27 cases (81.8%) with e-rILD and 60 cases (76.9%) without e-rILD began subsequent therapy. The best supportive care alone was selected in 3 cases (9.1%) with e-rILD and 14 cases (17.1%) without e-rILD (Table 2). The choices of subsequent therapies succeeding everolimus were generally similar between the 2 groups (Table 3). Chemotherapy was selected in 20 cases (74.1%) with e-rILD and 43 cases (71.6%) of patients without e-rILD. Among chemotherapy regimens, S-1 was the regimen of choice in approximately 40% of cases, taxane in approximately 20%, and capecitabine or anthracycline in the remaining cases (Table 3). Endocrine therapy alone was selected in 4 cases (14.8%) with ILD and 10 cases (16.7%) without e-rILD. Endocrine therapy plus CDK4/6 inhibitor was selected in 2 cases (7.4%) with e-rILD and 4 cases (6.7%) without e-ILD.

**Table 2:** Post-everolimus outcome.

	Patients treated with everolimus				
	With e-rILD (*n* = 33)		Without e-rILD (*n* = 82)		*P*-value
	*n*	%	*n*	%	
**Everolimus termination**	33	100	78	95.1	.197
** Start the next regimen**	27	81.8	60	76.9	.567
** Continue the same ET alone**	2	6.1	0	0	
** Best supportive care alone**	3	9.1	14	17.9	
** Unknown**	1	3.0	4	5.4	
**Everolimus continuation with another ET**	0	0	4	4.9	

Abbreviations: e-rILD, everolimus-related interstitial lung disease; ET, endocrine therapy; *n*, number.

**Table 3:** Subsequent treatment regimen immediately after everolimus.

	Patients treated with everolimus								*P*-value
	With e-rILD (*n* = 27)				Without e-rILD (*n* = 60)				
	*n*		%		*n*		%		
**Chemotherapy**	20		74.1		43		71.6		.816
** S-1**		12		44.4		23		38.3	
** Taxane**		5		18.5		12		20.0	
** Anthracycline**		1		3.7		1		1.7	
** Capecitabine**		1		3.7		1		1.7	
** Other**		1		3.7		6		10.0	
**Endocrine therapy**	4		14.8		10		16.7		.828
** Fulvestrant**		3		11.1		6		10.0	
** Tamoxifen**		1		3.7		2		3.3	
** Letrozole**		0		0		1		1.7	
** Medroxyprogesterone acetate**		0		0		1		1.7	
**CDK4/6 inhibitor**	2		7.4		4		6.7		
** Palbociclib + endocrine therapy**		2		7.4		3		5.0	
** Abemaciclib + endocrine therapy**		0		0		1		1.7	
**Clinical trial**	1		3.7		3		5.0		

Abbreviations: CDK, cyclin-dependent kinase; e-rILD, everolimus-related interstitial lung disease; *n*, number.

**Figure 1:**
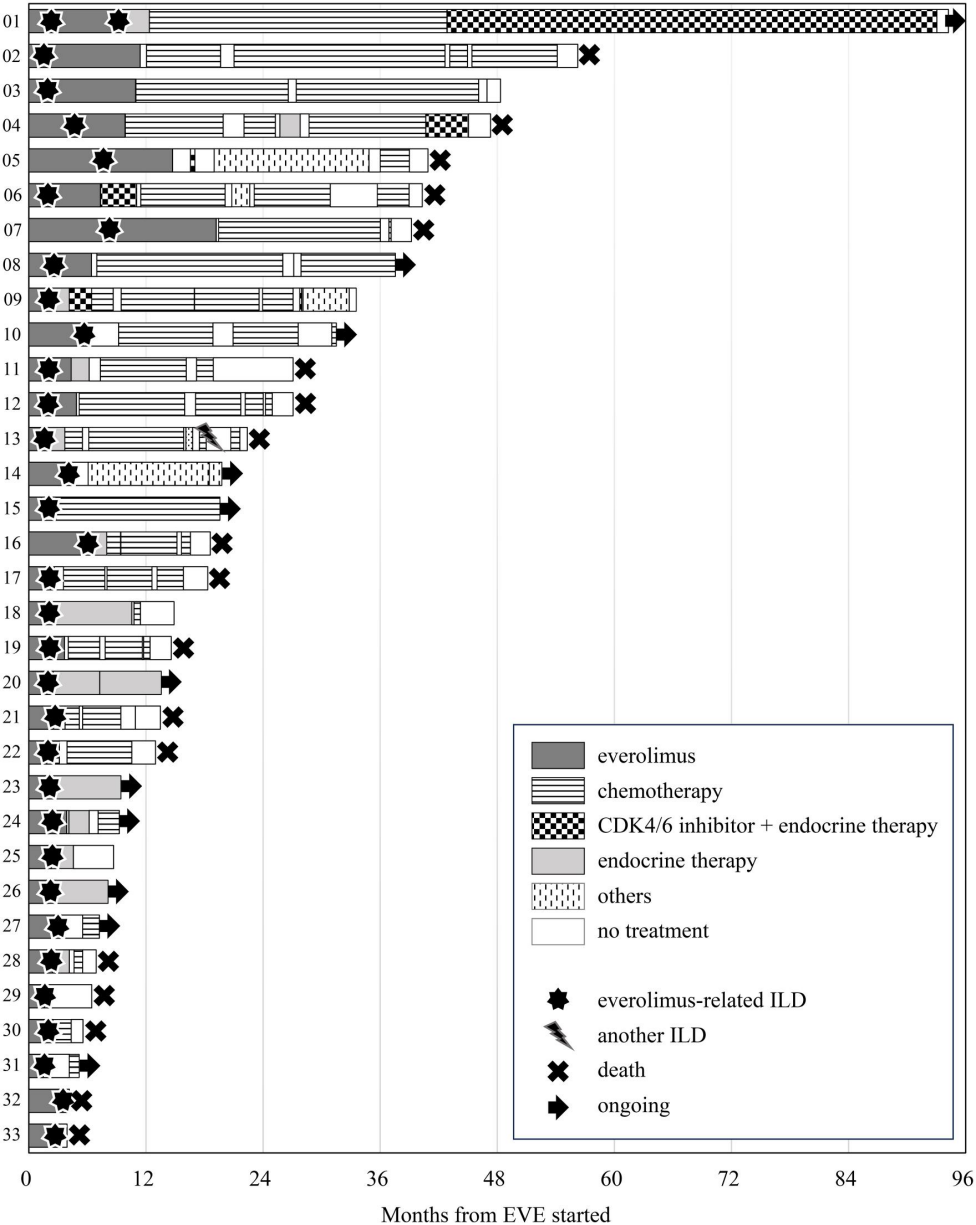
Treatment course of patients with everolimus-related interstitial lung disease.

**Figure 2:**
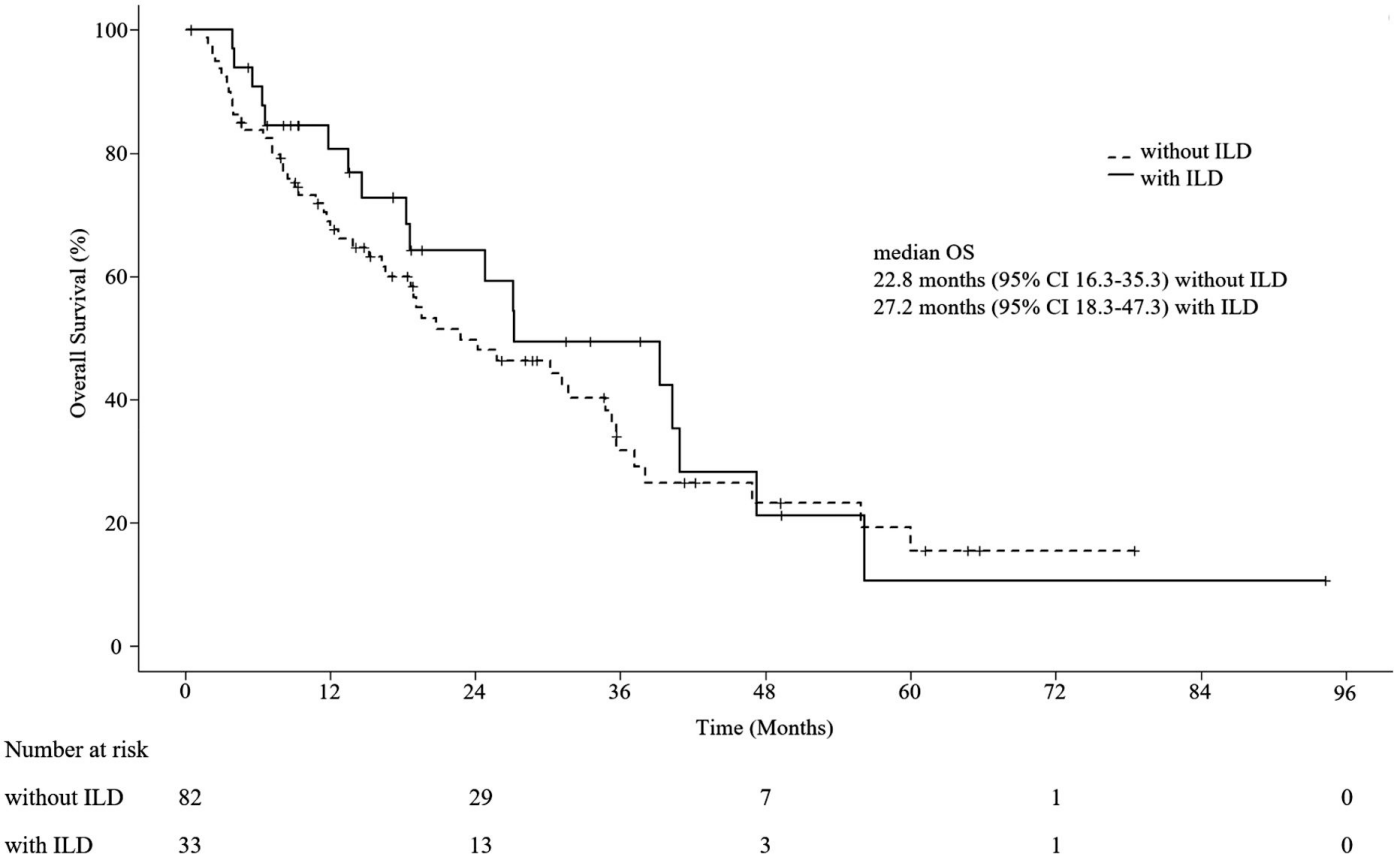
Kaplan–Meier curves comparing overall survival of patients with and without everolimus-related interstitial lung disease.

Although most cases were heavily treated and survival tended to be short, 18 of 33 (54.5%) patients were able to continue subsequent treatments for more than 1 year after e-rILD (Figure 1). The patient who twice experienced e-rILD was able to continue subsequent treatments for almost 7 years (case 1 in Figure 1). The median OS from the start of everolimus was 27.2 months (95% CI, 18.3-47.3) in cases with e-rILD and 22.8 months (95% CI, 16.3-35.3) in cases without e-rILD (Figure 2) (hazard ratio, 0.81; 95% CI, 0.47-1.38; *P* = .431). The median TTF was 4.8 months (95% CI, 3.0-6.0) for patients without e-rILD and 4.6 months (95% CI, 3.5-5.7) for those with e-rILD (HR, 1.11; 95% CI, 0.73-1.70; *P* = .630).

Among patients who received subsequent systemic therapy after everolimus, the median OS from subsequent therapy initiation was 22.7 months (95% CI, 14.8-37.5) for cases with e-rILD and 20.7 months (95% CI, 13.3-29.6) for those without e-rILD (HR, 0.87; 95% CI, 0.48-1.60; *P* = .669) (Figure S1).

### Drug-related ILD during post-everolimus therapies

At the data cut-off, patients with and without e-rILD received a median of 3 and 2 lines of post-everolimus therapy, respectively. During post-everolimus therapy, one case (3.7%) with e-rILD and 2 cases (3.3%) without e-rILD developed drug-related ILD (OR, 1.12; 95% CI, 0.12-12.86, *P* = .930). One patient developed grade 2 e-rILD and developed drug-related ILD while being treated with anthracycline as the fourth-line treatment after everolimus (case 13 in Figure 1).

## Discussion

Our study found that 28.7% of patients who received everolimus experienced e-rILD, with the majority classified as grade 1 or 2, and subsequently recovered with appropriate management. Most patients who experienced e-rILD could continue active subsequent anti-cancer therapies, and their choices of post-everolimus therapy were comparable to those of patients without e-rILD. Out of 33 patients with e-rILD, only 1 (3.0%) experienced another case of ILD caused by subsequent therapy, which is similar to the incidence seen in patients without e-rILD (2.4%).

The incidence of e-rILD was higher in our study, 28.7%, than in previous studies. In the BOLERO-2 study, the incidence of e-rILD was 16% in the overall population.^3^ The expanded-access multicenter phase IIIb trial in Europe (the BALLET study) reported treatment outcomes for 2,131 patients receiving exemestane plus everolimus. The incidence of non-infectious pneumonitis was 11.2% in patients over 70 years old and 8.9% in patients under 70, with no significant increase in age-related incidence.^11^ The BOLERO-2 study, however, suggested ethnicity-based differences in the incidence of e-rILD, with a higher incidence of 23.5% in the Asian population. All patients in our study were Japanese and the median age was >5 years higher than that of the Asian subset from the BOLERO-2 trial, which may have affected our findings.^10^ Additionally, our retrospective assessment of ILD, which included all suspected cases despite considering other differential diagnoses such as infectious pneumonia, may have contributed to the high incidence we observed.

The incidence of ILD associated with most cytotoxic chemotherapeutic drugs used in breast cancer treatment has been reported to be <1%: 0.3% for S-1, 0.5% for paclitaxel, and 0.4% for docetaxel, with unknown rates for capecitabine and doxorubicin.^12–14^ In contrast, the incidence of ILD associated with molecularly targeted drugs other than everolimus varies widely by drug. The incidence for CDK4/6 inhibitors is reported as 0.3%-2.1%, while the incidence for the antibody–drug conjugate trastuzumab deruxtecan is 15.8%, and those for immune checkpoint inhibitors (PD-1/PD-L1 inhibitors) range from 1.1% to 3.6%.^15^^,^^16^ It should be noted that these incidences are based on clinical trials, which in many cases excluded patients with preexisting ILD and those who had experienced ILD related to prior anti-cancer therapies. The choice of subsequent therapies for patients who experienced drug-related ILD is challenging for oncologists and no specific guidelines are available.

In our study, S-1 was most frequently chosen as the subsequent therapy immediately after everolimus, followed in descending order by taxanes, fulvestrant, and CDK4/6 inhibitors (Table 3). It is noteworthy that these trends were similar regardless of e-rILD status. S-1 has a relatively favorable safety profile for ILD. A retrospective analysis reported that in metastatic pancreatic cancer, even in patients who developed ILD associated with gemcitabine-based regimens, no cases of ILD relapse were observed with S-1 monotherapy.^17^ We originally expected a difference in choice of subsequent therapy between those with and without e-rILD, with S-1 preferred for the e-rILD group. However, in reality, there were no differences in the proportion of patients receiving subsequent S-1 treatment or in treatment preferences between the groups (Table 3). Differences in typical clinical scenarios between the groups and termination of everolimus with e-rILD vs progressive disease with everolimus likely affected the results.

e-rILD differs from drug-induced ILD caused by other agents in that grade 1 ILD may improve even while continuing everolimus.^18^ In fact, in our cohort, there was a case in which everolimus treatment was continued with improvement; in about one-third of cases, therapy was restarted. This suggests that e-rILD may have a different clinical course than ILD caused by other agents. Therefore, the findings of this study cannot be extrapolated to patients who develop ILD while on other agents. For example, with trastuzumab deruxtecan, there is pooled analysis data indicating that re-administration is manageable only after recovery from grade 1 ILD.^19^ Rechallenge with immune checkpoint inhibitors may be offered in patients with toxicity once recovered to grade <1,^20^ indicating that management strategies vary depending on the drug.

This study had several limitations. This was a single-center, retrospective study. Only approximately half of the cases received CDK4/6 inhibitors before everolimus, which may not fully reflect the current and evolving treatment landscape. In addition, no patients in our cohort received antibody–drug conjugates, including trastuzumab deruxtecan or datopotamab deruxtecan, as prior treatment before everolimus, as it was not indicated until after the data cut-off for this study. In addition, detailed information on comorbidities and concomitant medications was not systematically available for all patients, particularly those without e-rILD, because of the retrospective nature of this study, which may have resulted in residual confounding.

## Conclusions

e-rILD appeared to be more common in this Japanese cohort than previously reported. The majority of cases were mild and the patients recovered with appropriate management. The choice of treatment regimen following everolimus plus endocrine therapy and the incidence of drug-related ILD were comparable between patients with e-rILD and those without. The findings of this study suggest that active standard treatments after e-rILD may be feasible without a significantly increased risk of ILD relapse as long as cases are carefully selected. Further large-scale multicenter studies, including those evaluating everolimus-based combination regimens, are warranted to validate these observations and to clarity the safety of subsequent treatment strategies in this setting.

## Supplementary Material

oyag082_Supplementary_Data

## Data Availability

The data underlying this article will be shared upon reasonable request to the corresponding author.
