# Supplementary material for: Impact of everolimus-related interstitial lung disease on subsequent treatment in patients with metastatic breast cancer
Source: Oncologist. 2026 Mar 14;31(4):oyag082. doi: 10.1093/oncolo/oyag082 (PMC13010270; doi:10.1093/oncolo/oyag082)
Supplement: oyag082_Supplementary_Data [file oyag082_supplementary_data.zip › S1 figure legend.docx]

Fig. S1

Kaplan–Meier curves comparing overall survival from subsequent therapy initiation among patients who received subsequent systemic therapy after everolimus, stratified by the presence of everolimus-related interstitial lung disease.
