# Supplementary figures and images for: Impact of everolimus-related interstitial lung disease on subsequent treatment in patients with metastatic breast cancer
Source: Oncologist. 2026 Mar 14;31(4):oyag082. doi: 10.1093/oncolo/oyag082 (PMC13010270; doi:10.1093/oncolo/oyag082)

## Slide 1
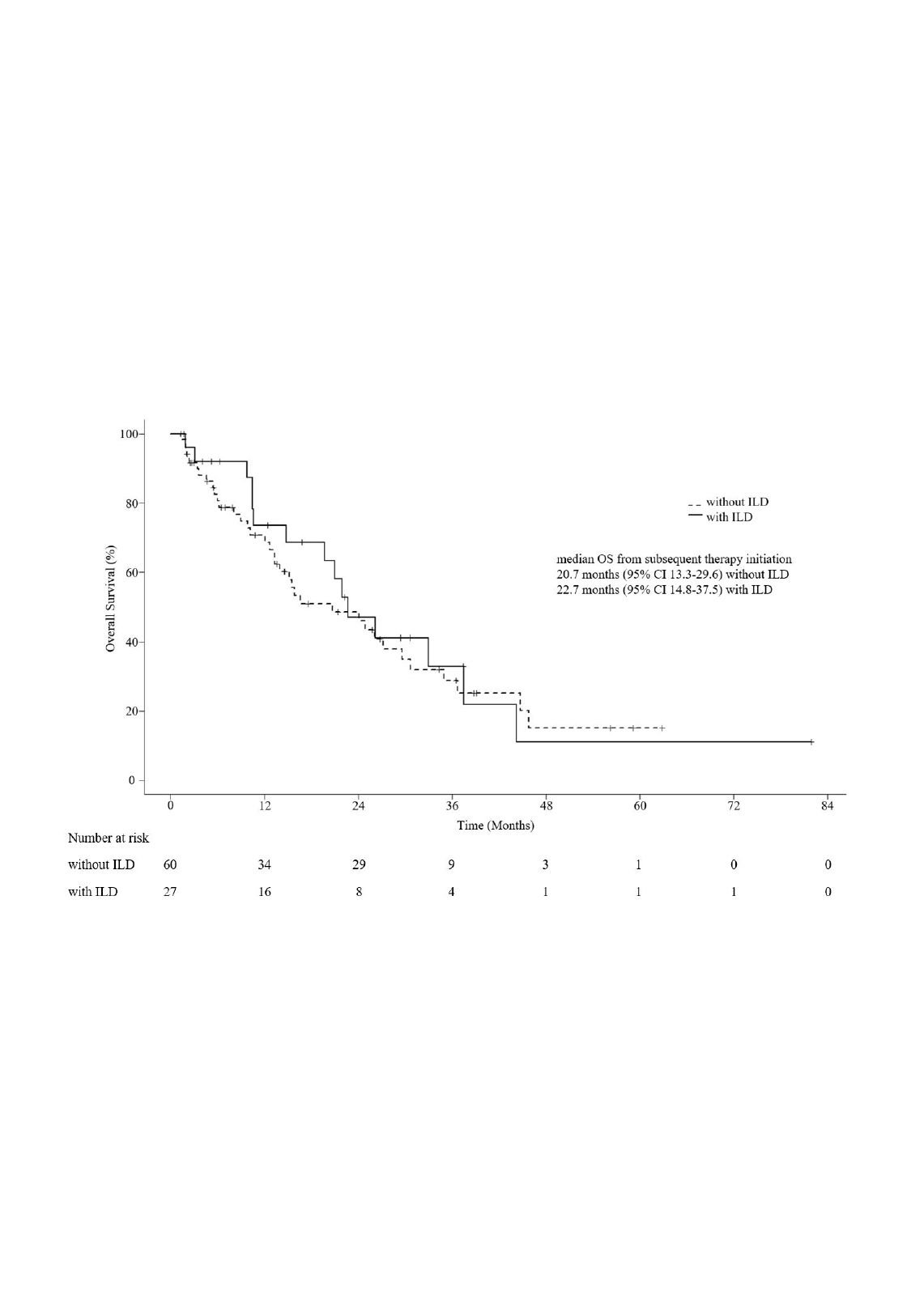

Supplement: oyag082_Supplementary_Data [file oyag082_supplementary_data.zip › figureS1.pptx]
